# Looking at Current Practices Regarding Implementation of Covert Administration of Medication Policy

**DOI:** 10.1192/bjo.2022.451

**Published:** 2022-06-20

**Authors:** Rehana Kauser, Mahmoud Saeed, Edward Marson, Saman Asad, Amoune Mohamed, Georgina Knowles

**Affiliations:** 1 Birmingham and Solihull Mental Health NHS Foundation Trust, Birmingham, United Kingdom; 2 University Hospitals Birmingham NHS Foundation Trust, Birmingham, United Kingdom

## Abstract

**Aims:**

The aims of the audit was to find out current practices regarding implementation of covert administration policy guidance. The Covert Medication Administration policy was introduced during the past two years, but due to ongoing pandemic, awareness of it was low. Guidelines for when making a decision to administer medication covertly were clear in the policy. Covert medication administration is a very restrictive practice, albeit clearly in a patient's best interests. Instances were found when medication for physical health was administered covertly and there isn't authority to do so under the Mental Health Act as noted in Care Quality Commissioning inspections.

**Methods:**

The sample selection was obtained by Incident Reporting forms for covert medication prescription from which 10 patients were identified from a four month retrospective sample of geriatric psychiatric inpatient admissions at the Juniper Centre at Moseley Hall Hospital, Birmingham from April to August 2021.

**Results:**

Covert medications administered were used to treat physical and mental health conditions. The physical health medication given was not for side-effects of mental health medication. Of the 22 medications and 10 patients there were no instances where the covert medication checklist had been completed. 9 of 22 medications (41%) (across 7 patients (70%)) had neither a best interest meeting nor a separate discussion held with the patient's family, friend, carer or advocate documented on the electronic record. Of the 22 medications, 7 medications (32%) belonging to 3 different patients had documentation of pharmacist involvement in the decision of covert medication administration whereas 15 medications belonging to 8 different patients did not.

**Conclusion:**

Our findings conclude inadequate following of the standards protocol of the covert medication administration policy. Despite 77% of medications being prescribed with a completed multi-disciplinary covert care plan and 95% of medications having had completed Incident Reporting forms, the rest of the standards were notably missed.

